# Protective Immunity Against *Neospora caninum* Infection Induced by 14-3-3 Protein in Mice

**DOI:** 10.3389/fvets.2021.638173

**Published:** 2021-03-03

**Authors:** Shan Li, Nan Zhang, Shaoxiong Liu, Jianhua Li, Li Liu, Xiaocen Wang, Xin Li, Pengtao Gong, Xichen Zhang

**Affiliations:** ^1^Key Laboratory of Zoonosis Research, Ministry of Education, College of Veterinary Medicine, Jilin University, Changchun, China; ^2^Department of Social Medicine and Public Health, School of Basic Medicine, Jiujiang University, Jiujiang, China; ^3^College of Basic Medicine, Jilin University, Changchun, China

**Keywords:** *Neospora caninum*, 14-3-3, extracellular vesicles, cytokines, immunity

## Abstract

*Neospora caninum* is an apicomplexan parasite that infects many mammals and remains a threatening disease worldwide because of the lack of effective drugs and vaccines. Our previous studies demonstrated that *N. caninum* 14-3-3 protein (Nc14-3-3), which is included in *N. caninum* extracellular vesicles (NEVs), can induce effective immune responses and stimulate cytokine expression in mouse peritoneal macrophages. However, whether Nc14-3-3 has a protective effect and its mechanisms are poorly understood. Here, we evaluated the immune responses and protective effects of Nc14-3-3 against exposure to 2 × 10^7^ Nc-1 tachyzoites. Antibody (IgG, IgGl, and IgG2a) levels and Th1-type (IFN-γ and IL-12) and Th2-type (IL-4 and IL-10) cytokines in mouse serum, survival rates, survival times, and parasite burdens were detected. In the present study, the immunostimulatory effect of Nc14-3-3 was confirmed, as it triggered Th1-type cytokine (IFN-γ and IL-12) production in mouse serum 2 weeks after the final immunization. Moreover, the immunization of C57BL/6 mice with Nc14-3-3 induced high IgG antibody levels and significant increases in CD8^+^ T lymphocytes in the spleens of mice, indicating that the cellular immune response was significantly stimulated. Mouse survival rates and times were significantly prolonged after immunization; the survival rates were 40% for Nc14-3-3 immunization and 60% for NEV immunization, while mice that received GST, PBS, or blank control all died at 13, 9, or 8 days, respectively, after intraperitoneal *N. caninum* challenge. In addition, qPCR analysis indicated that there was a reduced parasite burden and diminished pathological changes in the mice immunized with Nc14-3-3. Our data demonstrate that vaccination of mice with Nc14-3-3 elicits both cellular and humoral immune responses and provides partial protection against acute neosporosis. Thus, Nc14-3-3 could be an effective antigen candidate for vaccine development for neosporosis.

## Introduction

*Neospora caninum* is an intracellular protozoan parasite belonging to the phylum Apicomplexa and is the causative agent of Bovine neosporosis (1). Although there is no evidence that *N. caninum* infection occurs in humans, anti-*N. caninum* antibodies have been detected in humans (2), suggesting that it might be a potential zoonotic pathogen. Neosporosis can spread by transplacental transmission from an infected dam to her fetus and brings enormous economic losses to the cattle industry worldwide (3). However, there are no effective drugs or vaccines available to control this disease (4), and developing a potent vaccine against neosporosis is vital.

The 14-3-3 protein family includes highly conserved proteins that are widely expressed in all eukaryotic cells and are involved in many cellular processes, such as cell cycle control, signal transduction, protein trafficking, and responses to environmental stimulation (5). Furthermore, as 14-3-3 proteins have been indicated to be highly immunogenic (6), these proteins from parasites represent a rational approach for the development of effective vaccines against the respective infections. *T. gondii* 14-3-3 (Tg14-3-3) was detected in tachyzoites and found to be present mainly in the cytoplasm as well as within the parasitophorous vacuolar space, inducing the migratory activation of immune cells (7). Our previous research demonstrated that *N. caninum* 14-3-3 protein was included in *N. caninum* extracellular vesicles (NEVs), and the protein was mainly found in the cytosol and cell membrane in *N. caninum* tachyzoites and has shown that the *N. caninum* 14-3-3 protein can induce effective immune responses and stimulate cytokine expression by activating the MAPK, AKT, and NF-κB signaling pathways (8); however, the protective efficacy of 14-3-3 protein as a vaccine antigen against *N. caninum* remains unclear. Here, we purified a recombinant fusion Nc14-3-3 protein to assess its protective efficacy against *N. caninum* infection.

## Materials and Methods

### Animals and Parasites

Female C57BL/6 mice (6–8 weeks old) were purchased from the Changsheng Experimental Animal Center (Benxi, China). The mice were housed in isolator cages under specific pathogen-free conditions in the animal house of the Laboratory Animal Center of Jilin University (Changchun, China), and the food and water provided were sterile. All animal experimental procedures were performed in strict accordance with the Regulations for the Administration of Affairs Concerning Experimental Animals approved through the State Council of the People's Republic of China (1988.11.1) and with the approval of the Animal Welfare and Research Ethics Committee at Jilin University.

*N. caninum* tachyzoites (Nc-1 strain) were maintained by serial passage in Vero cells in RPMI-1640 medium, and free tachyzoites were obtained and harvested from Vero cells as described in a previous study (8, 9).

### *N. caninum* EV Preparation

NEVs were purified as previously described (9), and free tachyzoites were collected using Percoll and cultured in exosome-depleted culture medium for 24 h. The NEVs were collected by differential centrifugation. Briefly, the supernatant was first centrifuged at 300 × g for 10 min at 4°C, and then the supernatant was centrifuged at 2,000 × g for 10 min at 4°C and at 10,000 × g for 30 min at 4°C to remove the parasites and debris. Finally, the supernatant was further ultracentrifuged at 100,000 × g for 70 min at 4°C to precipitate the expected NEVs with an ultracentrifuge (Hitachi Micro Ultracentrifuge, Japan). The observed NEV pellets in each tube were collected together and ultracentrifuged once more at 100,000 × g for 70 min at 4°C. The final pellet was resuspended in cold PBS, and protein concentrations were measured using a BCA Protein Assay Kit (Thermo Scientific, Waltham, USA).

### Expression and Purification of Recombinant Nc14-3-3 Protein

The recombinant protein Nc14-3-3 was expressed as a glutathione S-transferase (GST) fusion protein in the *E. coli* expression strain Rosetta DE3a (TIANGEN, Beijing, China). After induction of expression, the recombinant proteins were purified using Proteinlso^®^ GST Resin (TransGen Biotech, Beijing, China) as described previously (8). The induced expression of the pGEX-4T-1 empty vector and purified GST-tagged protein were used as controls.

### Mouse Immunization and Challenge

To assess the immunogenicity of Nc14-3-3, female C57BL/6 mice were randomly divided into five groups (16/group) as follows: Blank, PBS, GST, NEVs, and Nc14-3-3. NEVs were dissolved in sterile PBS to a final concentration of 1 μg/μl, and mice were injected intramuscularly with 50 μl of NEVs or PBS alone. Otherwise, mice were intramuscularly immunized with recombinant Nc14-3-3 protein or GST protein (50 μg) emulsified with Freund's adjuvant (Sigma, St. Louis, USA). Briefly, mice were intramuscularly immunized with Nc14-3-3 protein or GST protein, which was emulsified with Freund's complete adjuvant (Sigma, St. Louis, USA). Boosters were administered at 2, 4, and 6 weeks, and the proteins were emulsified with Freund's incomplete adjuvant (Sigma, St. Louis, USA). Two weeks after the third immunization, each mouse was challenged with a dose of 2 × 10^7^ Nc-1 tachyzoites, and the survival time, body weight and clinical observations of the mice (*n* = 10) were observed and recorded every day by the same person at similar time points.

### Determination of Serum Antibody and Cytokine Levels

Mouse polyclonal antibodies were prepared as follows: serum was collected from the mice via the tail veins on the day before each vaccination. The antibody levels in mouse serum was detected by indirect enzyme-linked immunosorbent assays (ELISA) as previously described (10). Briefly, plates were coated with 2 μg of *N. caninum* lysate antigen (NLA), and mouse sera diluted in PBST at 1:100 were added. HRP-labeled antibodies (IgG, IgG1, or IgG2a, 1:2,000 dilution) (Proteintech, Wuhan, China) were used as secondary antibodies. The reaction was detected by TMB (Beyotime, Shanghai, China) and stopped by 2 M H_2_SO_4_ addition. Absorbance at 450 nm was measured with a microplate reader. In addition, NLA was prepared as previously described (9). Briefly, *N. caninum* tachyzoites were resuspended in BAG buffer (116 mM NaCl, 5.4 mM KCl, 0.8 mM MgSO4, 50 mM HEPES, 5.5 mM d-glucose, pH 7.3) with protease inhibitors (KeyGen Biotech, Nanjing, China) and subjected to ultrasonic disruption (60 Hz/30 s) on ice. After centrifugation at 10,000 × g for 30 min at 4°C, the NLA was collected and filtered using 0.22 μm membranes. The protein concentrations were measured using the BCA Protein Assay Kit (Thermo Scientific, Waltham, MA, USA).

To evaluate the concentration of cytokines in serum samples, 2 weeks after the final immunization, six mice were sacrificed, blood was collected from the eyeball, and serum was obtained for cytokine measurements. Cytokine ELISA Ready-SET-Go kits (eBioscience, San Diego, CA, USA) were used to detect the IL-12p40, IFN-γ, IL-10, and IL-4 levels according to the manufacturer's instructions.

### Flow Cytometry Analysis of T Cell Subpopulations

The percentages of T cell subpopulations in the spleens of mice in the experimental groups were detected by flow cytometry. The spleens were obtained 2 weeks after the final immunization from mice (*n* = 6) in each group, and a flow cytometry assay was performed as previously described (11). Briefly, 1 × 10^6^ splenocytes were suspended in pre-cooled PBS and incubated with anti-mouse CD3-PerCP, anti-mouse CD4-PE, and anti-mouse CD8-APC antibodies (all from BioLegend) at 4°C for 30 min in the dark. Then, the cells were washed twice with pre-cooled PBS, resuspended in PBS and analyzed with a FACSAria flow cytometer (BD Biosciences), with 20,000 total events/sample. Data were analyzed by FlowJo software (Tree Star Inc.).

### Quantification of the Parasite Burden by qPCR

Two weeks after the last immunization, each mouse was challenged with 2 × 10^7^ Nc-1 tachyzoites. At 5 days post-infection, infected mice were euthanized, and the heart, liver, spleen, lung, kidney, and brain were harvested and stored at −40°C. The parasite replication in tissues were monitored by real-time quantitative PCR (qPCR) as previously described (12). Briefly, the tissues were homogenized and used for DNA extraction (TIANGEN, Beijing, China). Five hundred nanograms of extracted DNA from each sample was amplified with the Nc5 sequence of *N. caninum* (forward: 5′-ACTGGAGGCACGCTGAACAC-3′, reverse: 5′-AACAATGCTTCGCAAGAGGAA-3′) using FastStart Universal SYBR Green Master Mix. To quantify the number of parasites, a standard curve was generated by amplifying 10-fold dilutions of 2.3 × 10^8^ *N. caninum* tachyzoites in separate reactions.

### Histopathology

Pathological changes were observed by H&E staining, and fresh tissue was fixed with 10% neutral buffered formalin and routinely processed in paraffin. Fixed paraffin-embedded tissues were sectioned at 3–4 μm and stained with haematoxylin and eosin (H&E).

### Statistical Analysis

Statistical significance was determined by one-way ANOVA using SPSS 19.0 software (SPSS Inc., Chicago, IL, USA), and data are shown as the mean ± standard deviation (SD) of triplicate experiments. GraphPad Prism 7 (GraphPad Software, Inc., San Diego, CA, USA) was utilized to generate the graphs. *P* < 0.05 were considered significant.

## Results

### Serum Antibody Responses in C57BL/6 Mice

**Figure 1:**
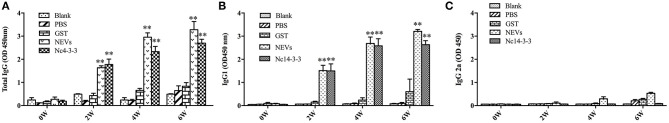
Measurement of specific IgG antibodies in the sera of immunized mice. Mouse serum was collected from the tail vein plexus before each vaccination, and antibodies were detected by indirect enzyme-linked immunosorbent assays (ELISAs). Determination of specific IgG antibodies in the serum at 0, 2, 4, and 6 weeks. **(A)** Total IgG; **(B)** IgG1; and **(C)** IgG2a. The results are shown as the means of OD450 ± SDs, and significant differences compared with PBS or GST (^**^P < 0.01) is indicated by asterisks (^*^).

To assess changes in antibody levels caused by recombinant Nc14-3-3 protein, the total IgG antibody level and the distribution of IgGl and IgG2a isotype 2 were tested after each immunization. As shown in Figure 1, significantly higher levels of IgG antibodies were observed in the groups vaccinated with NEVs and Nc14-3-3. In contrast, specific higher IgG1 antibody levels were detected in the above mentioned groups, but IgG2a was not obviously induced. No or low detectable levels of antigen-specific antibodies were observed in the control group receiving PBS or GST, respectively. These results suggested that immunization with NEVs or Nc14-3-3 can induce Th2 immune responses against *N. caninum* in mice.

### Levels of Cytokines in the Sera of Vaccinated Mice

**Figure 2:**
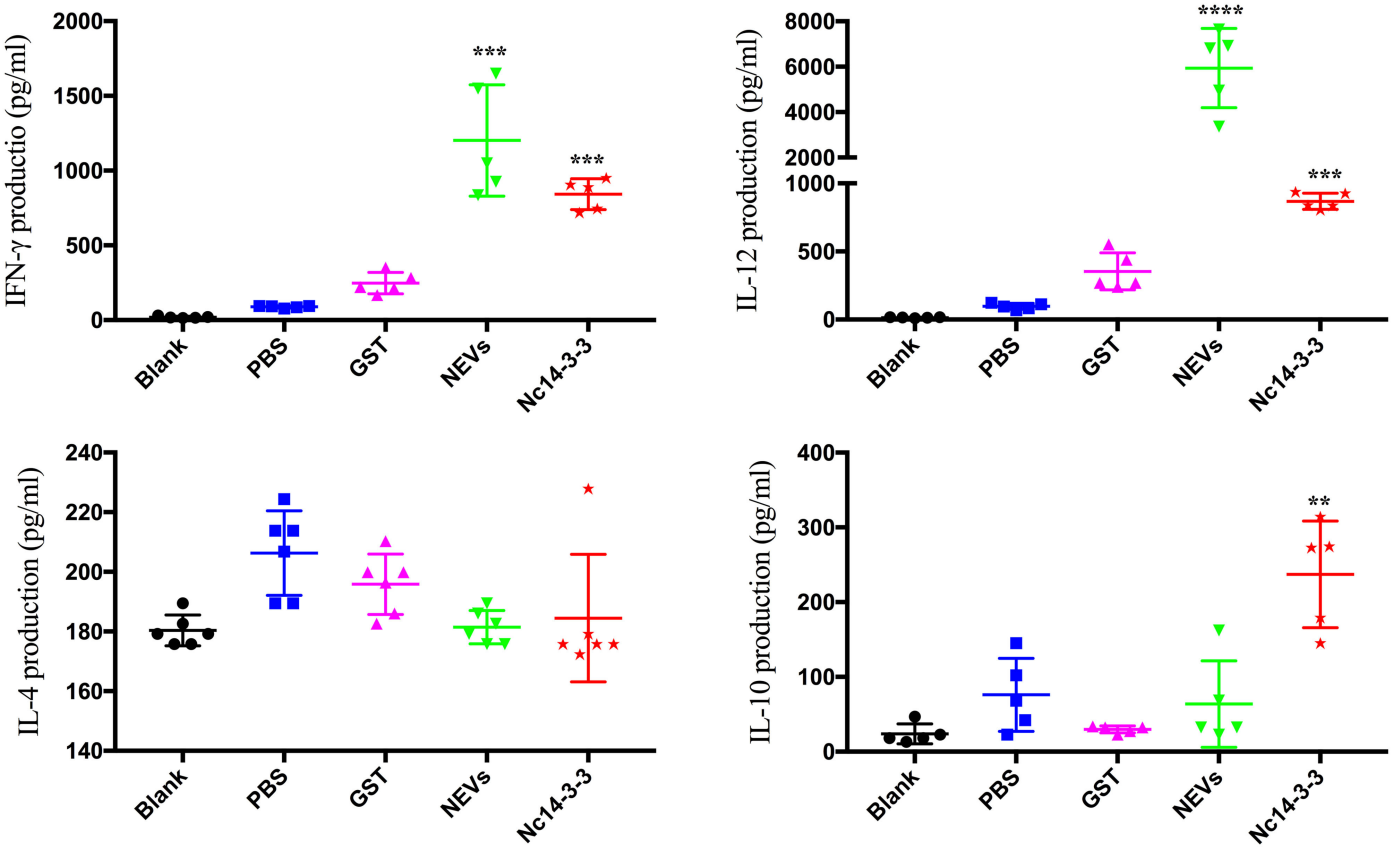
Cytokine production in the serum of mice detected using indirect enzyme-linked immunosorbent assays (ELISAs). Data are expressed as the mean ± SD from three separate experiments. ^*^P < 0.05; ^**^P < 0.01; ^***^P < 0.001; and ^****^P < 0.0001 for the NEV- and Nc14-3-3-immunized groups vs. the PBS or GST group.

To evaluate the cytokines released in serum samples in immunized groups, mice were sacrificed after the final immunization. As shown in Figure 2, mice vaccinated with NEVs or Nc14-3-3 generated significantly higher levels of IFN-γ and IL-12p40 than mice vaccinated with single-gene plasmids, PBS or empty vector (*P* < 0.01). In contrast, high IL-10 levels were observed in Nc14-3-3-immunized mouse serum compared with those in serum from the other groups (*P* < 0.01), and the levels of the cytokine IL-4 were not significantly different among all groups (*P* > 0.05). These results indicated that NEVs and Nc14-3-3 mainly cause Th1-type immune responses in mice, and the cytokines IFN-γ and IL-12 play an important role in protection against *N. caninum* infection after vaccination.

### Nc14-3-3 Immunization Increased CD8^+^ T Cell Levels

**Figure 3:**
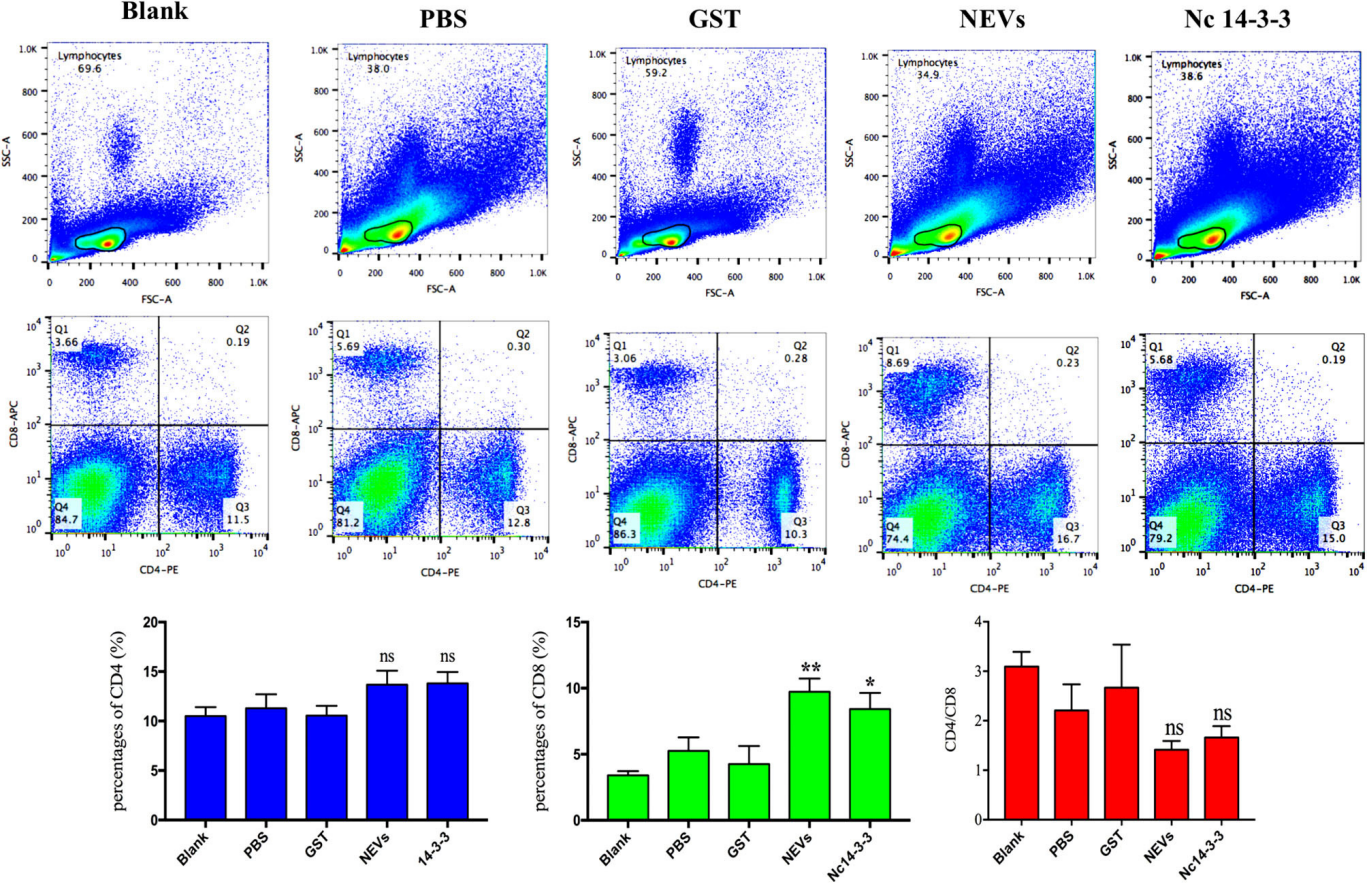
Flow cytometry analysis of T cell subsets. The percentages of CD4^+^ and CD8^+^ T cells in the spleen of immunized mice 2 weeks after the last immunization are shown, and the results are representative of three independent experiments. ^*^P < 0.05; ^**^P < 0.01 for the NEV- and Nc14-3-3-immunized groups vs. the PBS or GST group.

It is well-established that T cells play an important role in protective immunity against protozoan infections. To determine whether NEV or Nc14-3-3 vaccination activated CD4^+^ or CD8^+^ T cells, flow cytometry was used, and as shown in Figure 3, there was no significant difference in the percentage of CD4^+^ T cells in the mice immunized with different vaccines, but the percentage of CD8^+^ cells in the groups vaccinated with NEVs or Nc14-3-3 was significantly increased compared with that in the control groups that received PBS or GST (*P* < 0.05).

### Experimental Protection After *N. caninum* Infection in Mice

**Figure 4:**
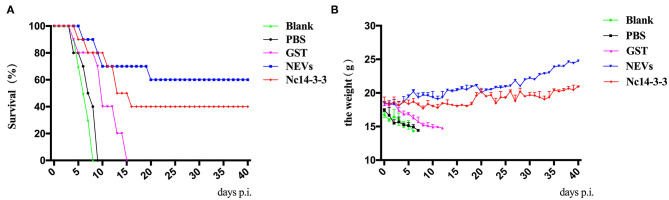
Survival rates and clinical observations of mice. **(A)** Survival rates (surviving mice/total mice) of vaccinated mice in response to infection with a dose of 2 × 10^7^ Nc-1 tachyzoites over 40 days. **(B)**. The body weight of mice was recorded daily before death occurred.

To assess the protection provided by NEVs or Nc14-3-3, 2 weeks after the last immunization, all mice were challenged with 2 × 10^7^ Nc-1 tachyzoites, and the survival time was monitored daily until 40 days after the challenge. Mice were highly susceptible to acute infection, and increased mortality was observed in the mice that received GST, PBS, or blank control (all of these mice died within 12, 8, or 7 days, respectively) (Figure 4A). In contrast, the survival rates for the NEV- or Nc14-3-3-immunized groups were 60 and 40% at the end of the trial, which showed significantly prolonged survival times. Furthermore, the body weight continuously decreased in the control groups vaccinated with PBS, GST or blank control until death, although no significant weight increase in NEV- or Nc14-3-3-immunized mice was found (Figure 4B).

### Vaccination With NEVs or Nc14-3-3 Controlled *N. caninum* Proliferation and Reduced Host Pathological Changes

**Figure 5:**
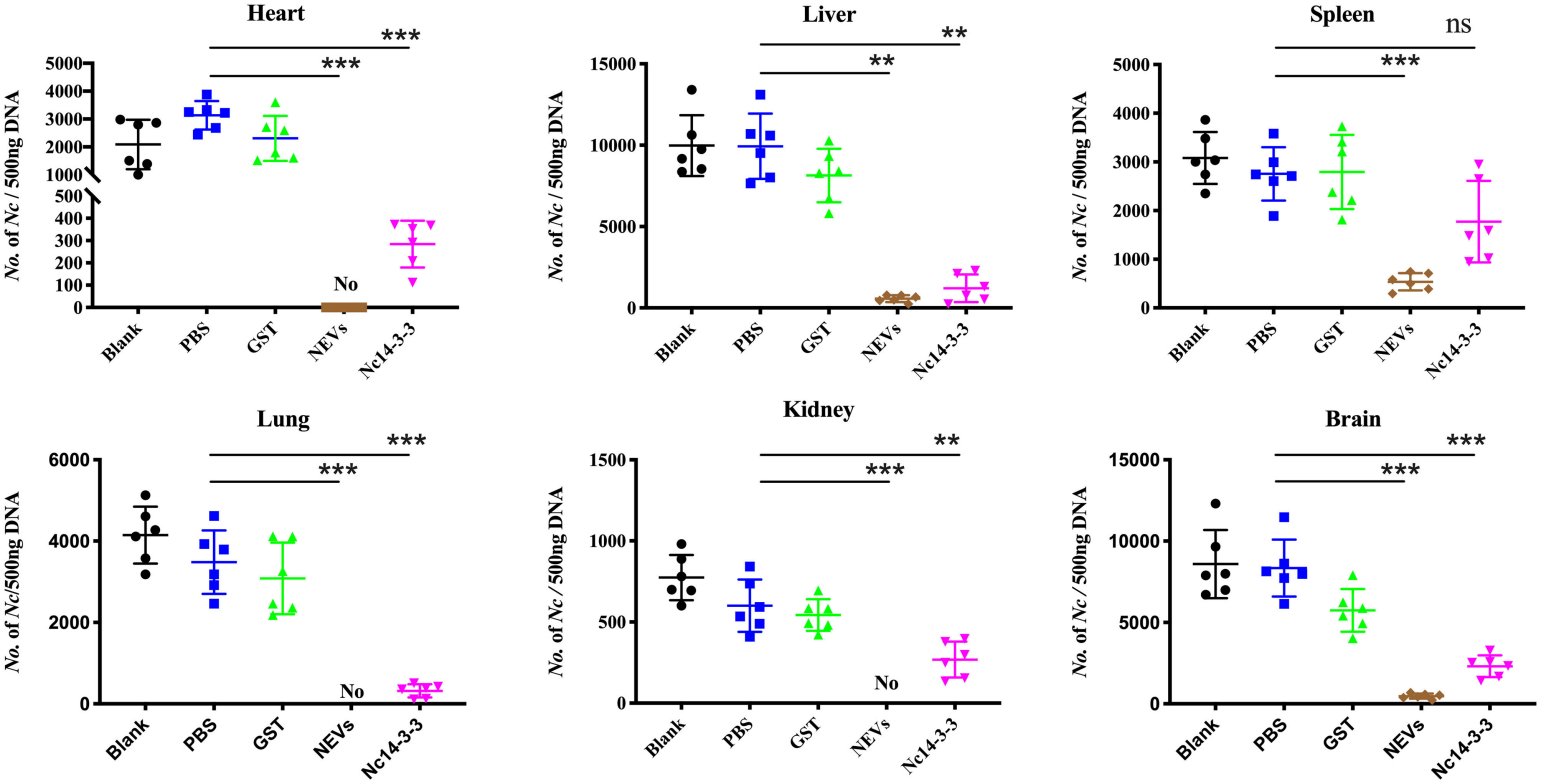
Two weeks after the last injection, each mouse was challenged with 2 × 10^7^ Nc-1 tachyzoites. At 5 days post-infection, infected mice were euthanized; the heart, liver, spleen, hung, kidney, and brain were harvested; and parasite loads were measured by qPCR. ^**^P < 0.01; and ^***^P < 0.001 for the NEV- and Nc14-3-3-immunized groups vs. the PBS or GST group.

**Figure 6:**
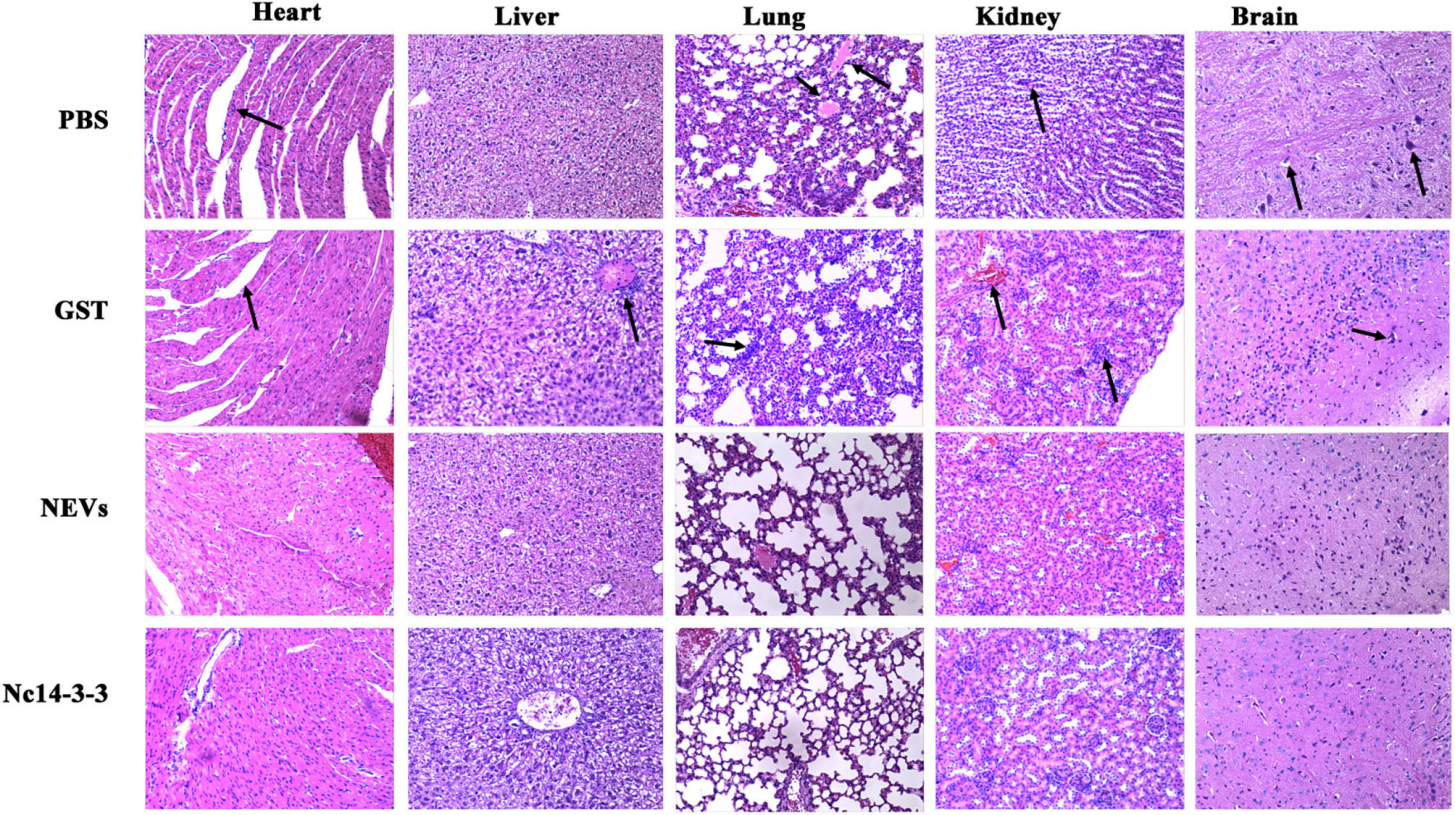
Two weeks after the last injection, each mouse was challenged with 2 × 10^7^ Nc-1 tachyzoites. At 5 days post-infection, infected mice were euthanized, and the heart, liver, hung, kidney, and brain were harvested. Pathological changes were observed by H&E staining.

At 5 days post-infection, infected mice were euthanized, and the heart, liver, spleen, lung, kidney, and brain were harvested to examine the parasite burden by qPCR and pathological changes by H&E staining. As shown in Figure 5, the number of parasites in the NEV- or Nc14-3-3-immunized group was significantly lower than that in the other groups vaccinated with PBS, GST or blank control (*P* < 0.05). The pathological changes shown in Figure 6 indicate that tissue lesions in the PBS and GST groups were serious, especially the thickening of the lung interstitium infiltrated with the most inflammatory cells, increased fluid in the alveoli, and widened alveolar septum. The liver structure was disordered and had necrotic foci, accompanied by a large number of inflammatory cells. The brain glial cells increased and exhibited macrophage infiltration, while the NEV- and Nc14-3-3-immunized mice had mild lesions. These results suggest that NEVs and Nc14-3-3 led to effective protection in the mouse model following infection with *N. caninum* tachyzoites.

## Discussion

*Neospora caninum* is an apicomplexan parasite that infects a broad range of warm-blooded animals and leads to neosporosis worldwide (13), and neosporosis often causes abortion especially in dairy cattle and leads to global economic losses (12).

Although many efforts have been made to restrain bovine neosporosis, there are still no effective methods to control this disease (14). Therefore, there is an urgent need to develop a safe and effective *N. caninum* vaccine (3). In recent years, with the identification of new antigens, several dense granule (GRA) and rhoptry (ROP) proteins in *N. caninum* have been identified that could be used in diagnostics or as vaccine candidates (15–17). We have previously demonstrated that the *N. caninum* 14-3-3 protein, which is included in extracellular vesicles (EVs) released by *N. caninum*, induced effective immune responses and stimulated cytokine expression through the MAPK, AKT and NF-κB signaling pathways in murine bone marrow-derived macrophages (BMDMs) (8, 9), but whether Nc14-3-3 can be used as a novel vaccine candidate against neosporosis has not yet been determined.

Currently, the most effective way to control neosporosis is to develop an effective vaccine. The first vaccines developed against *N. caninum* were live or attenuated vaccines because they could elicit both humoral and cellular immunity and provide a variable degree of protection, but their use was limited due to potential safety problems. Inactivated or classical subunit vaccines against neosporosis are safe but currently do not stimulate protective immunity (4). To date, numerous vaccine antigens have been widely evaluated, especially DNA, recombinant protein, or vector-based vaccines, and vaccination using a recombinant antigen triggering appropriate levels of protective immunity for effective protection could offer the most appropriate vaccination tool (15), such as surface proteins and/or those secreted from micronemes, rhoptries or dense granules, which have been the most studied for protection. The 14-3-3 protein is a phosphoserine-binding protein that plays a key role as a regulator of multiple cellular processes in eukaryotes (18, 19), and it has been isolated and sequenced in many apicomplexan parasites, such as *Toxoplasma gondii* (20), *Eimeria tenella* (21), and *Cryptosporidiidae* (22). More importantly, research has shown that 14-3-3 proteins can be used as vaccines in sheep infected with *Fasciola hepatica* (23). *Eimeria maxima* 14-3-3 could significantly reduce jejunal lesions and weight loss, increase the oocyst reduction ratio, and produce an anticoccidial index of more than 165, demonstrating that Em14-3-3 could be used as a promising antigen candidate for vaccine development against *E. maxima* (24). The 14-3-3 protein of *T. gondii* has been shown to be a new candidate vaccine against toxoplasmosis (25). Our previous research indicated that NEVs were enriched for secreted membrane-associated proteins, including 14-3-3, and EVs modulated inflammatory cytokine expression in BMDMs by triggering the TLR2 and MAPK signaling pathways *in vitro* (9). Increasing evidence has indicated that EVs can evoke the innate immune response to control or facilitate infection in parasites (26–28). EVs contain a variety of substances, including proteins, lipids, and RNA, that play multiple roles in intercellular communication, such as delivering signals, regulating cytokine secretion, and regulating the immune response, so EVs are novel vaccine candidates (10, 29, 30); therefore, our previous study also selected NEVs as vaccine controls.

When *N. caninum* invasion occurs, the parasite is first captured and processed by antigen-presenting cells and then presented to T lymphocytes to induce adaptive immunity. Upon subsequent invasion, specific antibodies, as one of the robust protective immune responses, can prevent and inhibit the attachment of *N. caninum* to its host cell receptors, further helping macrophages kill and eliminate the parasite and preventing reactivation (31–33). Humoral immunity is important in eliminating pathogens, strengthening the elimination of invasive microbes, and building immunological memory to protect against reinfection. In this study, we determined the humoral response on the basis of specific anti-*N. caninum* IgG levels; significantly increased levels of IgG were observed in the serum of mice vaccinated with NEVs and Nc14-3-3, which would contribute to strong protective efficacy against subsequent *N. caninum* infection. Higher levels of IgG1 were also detected in the serum of mice in the NEV- and Nc14-3-3-immunized groups than the serum of those in the control groups, and the level of IgG1 antibodies was significantly higher than that of IgG2a, indicating that NEVs and Nc14-3-3 induced a mixed Th1/Th2 immune response. These results are similar to those for other previously reported *T. gondii* vaccines (31, 34, 35). Type 1 immune responses are known to play an important role in protection against intracellular pathogens, and these responses are associated with high levels of IFN-γ and IL-12 (36). Therefore, we subsequently examined the expression of Th1 (IFN-γ and IL-12) and Th2 cytokines (IL-4 and IL-10) (37).

The elimination of intracellular protozoan parasites depends critically on the action of cellular immunity. Through interactions with many effector cells and molecules, a variety of immune cells actively cooperate to fight against infection. Accumulating evidence has shown that IL-12 triggers a Th1-type immune response, is a pivotal proinflammatory cytokine for the control and restriction of acute and chronic protozoan parasite infection, and is required for the long-term maintenance of the cytokine IFN-γ (38, 39). Notably, high levels of the IL-12 cytokine are essential to resistance against *N. caninum* and *T. gondii* infection (40, 41), and blocking or lacking a functional IL-12 receptor leads to high susceptibility to these two parasite infections (42). In addition, IL-12 is a well-known inducer of IFN-γ production in parasite infection, and studies have shown that IFN-γ and IL-12p40 are important to further explore the host protective mechanism. IL-12/IL-23 p40 chain-deficient (IL-12^−/−^) mice presented elevated parasitic burdens after intraperitoneal infection with *N. caninum* (43). Calves challenged with live tachyzoites could produce a predominant IgG response, and high levels of IFN-γ and TNF-α were also observed in animals (44, 45). Our study demonstrated that mice vaccinated with NEVs or Nc14-3-3 generated significantly higher levels of IFN-γ and IL-12p40 than mice vaccinated with single-gene plasmids, PBS or empty vector. These results suggested that immunization with NEVs or Nc14-3-3 elicited a Th1-type immune response against acute *N. caninum* infection. Furthermore, we also detected the levels of the Th2-type cytokines IL-4 and IL-10. High IL-10 levels were observed in Nc14-3-3-immunized mouse serum but not NEV-immunized mouse serum, and IL-4 levels did not change significantly in any group. This finding was similar to the results from BALB/c mice immunized with *T. gondii* exosomes (10). A large number of studies have shown that many proteins, including GAG1, ROP18, and GRA7, could protect mice after *T. gondii* infection by inducing the production of Th1-biased immune responses (46, 47). BALB/c mouse vaccination with the recombinant protein rNcSRS2 of *N. caninum* promoted the upregulation of IL-10 expression (48). Another study also reported that the IL-10 and IFN-γ cytokines were highly expressed in mouse spleen cells restimulated with *Neospora* antigens. IL-10 is a homodimeric cytokine, which can be produced by most cells of the innate and adaptive immune system, in addition, it functions as a self-limiting mechanism of effector T cells. During infection, it is produced to limit inflammation and collateral tissue damage, such as during *T. gondii* infection, IL-10 produced by Th1 cells is essential to limit an otherwise excessive and detrimental Th1 cell response (49). IL-10 is an essential anti-inflammatory cytokine that plays important roles as a negative regulator of immune responses (50); it is able to regulate the Th1-type response and promote high levels of IFN-γ production (51). Therefore, the high IL-10 level contributed to the prolonged survival time of mice immunized with Nc14-3-3.

Cellular immunity plays an important role in the control of *N. caninum* infection. To determine whether NEV or Nc14-3-3 vaccination activated CD4^+^ or CD8^+^ T cells, we determined the percentage of CD4^+^ and CD8^+^ T lymphocytes in the spleens of mice from each experimental group (*n* = 6) after the final immunization by flow cytometry. The results indicated that the percentage of CD8^+^ cells in the NEV- or Nc14-3-3-vaccinated mice was significantly increased, while there was no significant difference in the percentage of CD4^+^ T cells. These results were similar to those of Li's report, which showed that the percentage of CD8^+^ T cells was significantly increased in BALB/c mice immunized with *T. gondii* exosomes (10). IL-12 stimulates IFN-γ synthesis by natural killer (NK) cells, and T lymphocytes control intracellular replication of *Toxoplasma* (52). In the acute phase of infection, IFN-γ and IL-12, which involve CD8^+^ T cells, play critical roles in the detection and elimination of pathogens and, to a lesser extent, CD4^+^ T cells (53). CD8^+^ dendritic cells (DCs) are important *in vivo* for cross-presentation of antigens derived from intracellular pathogens (54), in the case of parasitic infection, CD8^+^ T cells are exposed to persistent antigen and/or inflammatory signals (55). CD8^+^ T cell contribute to the early production of the pro-inflammatory cytokine IL-12, which stimulates the release pro-inflammatory cytokine of IFN-γ, the generation of I pro-inflammatory is central to host resistance (56). As *T. gondii* is an intracellular parasite, a strong CD8^+^ T cell response plays an important role in controlling the development and spread of *T. gondii* infection (57). The current research indicated that mice immunized with NEVs and Nc14-3-3 activated specific cellular immunity against *N. caninum*.

Host innate immunity plays an important role in fighting protozoal infections by inhibiting parasite replication and triggering appropriate adaptive immune responses, which control active infections and overcome subsequent re-exposures (58). A number of mechanisms for how the immune system recognizes and responds to pathogens have been defined, including mechanisms regulated by EVs (59). EVs likely play an important role during parasite infection because of their diverse group of biomolecules, which have immunomodulatory properties (60, 61). As a vaccine, exosomes were associated with the development of fewer brain cysts in *T. gondii*-infected CBA/1 mice (62). *T. gondii* exosomes were also found to trigger the immune response and activate partial protective immunity against acute *T. gondii* infection in BALB/c mice (10). *Leishmania* exosomes have also been well-studied, and *Leishmania donovani* exosomes modulate innate and adaptive immune responses in C57BL/6 mice by affecting monocyte and dendritic cell (DC) cytokine production (63). *Leishmania major* releases exosomes with different protein contents, which are known to function in immune modulation (64). DCs pulsed with *Eimeria* antigens could be used as a vaccine against *Eimeria* infection in chickens (65). Mice treated with exosomes derived from DCs pulsed with *T. gondii* antigens were shown to elicit humoral and cellular immune responses and protect mice against subsequent parasite infection (66). Although the above results all indicated that EVs could be potential candidates, a variety of antigen components from pathogens can be loaded in EVs, and they will not cause infection due to the lack of live parasites. However, considering that obtaining high-purity EVs or exosomes is still difficult at present, much work needs to be done to identify and validate EV biomarkers that can be utilized in the diagnosis and therapy of parasitic disease.

## Conclusions

In the work described here, we examined the immunogenicity and potency of Nc14-3-3 as a vaccine candidate against infection with *N. caninum* in a murine model. Our data demonstrate that the vaccination of mice with Nc14-3-3 elicited both cellular and humoral immune responses and provided partial protection against acute neosporosis. Thus, Nc14-3-3 could be used as an effective antigen candidate for developing vaccines against *N. caninum*.

## Data Availability Statement

The original contributions presented in the study are included in the article/supplementary material, further inquiries can be directed to the corresponding author/s.

## Ethics Statement

The animal study was reviewed and approved by all animal experimental procedures were performed in strict accordance with the Regulations for the Administration of Affairs Concerning Experimental Animals approved by the State Council of People's Republic of China (1988.11.1) and with the approval of the Animal Welfare and Research Ethics Committee at Jilin University (IACUC Permit Number: 20160612).

## Author Contributions

SLi, NZ, and XZ developed the study protocol. SLi and SLiu carried out the experiments. SLi, XW, XL, and LL performed the data analysis. SLi wrote the manuscript. PG, JL, and XZ revised the manuscript. All authors read and approved the final manuscript.

## Conflict of Interest

The authors declare that the research was conducted in the absence of any commercial or financial relationships that could be construed as a potential conflict of interest.
